# Adaptive Resource Utilization Prediction System for Infrastructure as a Service Cloud

**DOI:** 10.1155/2017/4873459

**Published:** 2017-07-25

**Authors:** Qazi Zia Ullah, Shahzad Hassan, Gul Muhammad Khan

**Affiliations:** ^1^Computer Engineering Department, Bahria University, Islamabad, Pakistan; ^2^Department of Electrical Engineering, COMSATS Institute of Information Technology Attock, Attock, Pakistan; ^3^Department of Electrical Engineering, University of Engineering and Technology, Peshawar, Peshawar, Pakistan

## Abstract

Infrastructure as a Service (IaaS) cloud provides resources as a service from a pool of compute, network, and storage resources. Cloud providers can manage their resource usage by knowing future usage demand from the current and past usage patterns of resources. Resource usage prediction is of great importance for dynamic scaling of cloud resources to achieve efficiency in terms of cost and energy consumption while keeping quality of service. The purpose of this paper is to present a real-time resource usage prediction system. The system takes real-time utilization of resources and feeds utilization values into several buffers based on the type of resources and time span size. Buffers are read by R language based statistical system. These buffers' data are checked to determine whether their data follows Gaussian distribution or not. In case of following Gaussian distribution, Autoregressive Integrated Moving Average (ARIMA) is applied; otherwise Autoregressive Neural Network (AR-NN) is applied. In ARIMA process, a model is selected based on minimum Akaike Information Criterion (AIC) values. Similarly, in AR-NN process, a network with the lowest Network Information Criterion (NIC) value is selected. We have evaluated our system with real traces of CPU utilization of an IaaS cloud of one hundred and twenty servers.

## 1. Introduction

The demand for high performance computing has transformed the shape of today's computer industry. The computing is no more limited to personal computers and work stations. It has now become a public grid, where users (personal computers, cell phones, work stations, and servers) can have access to the storage and computing resources through internet. The environment where users can have access to a distant infrastructure, platform, or software over the internet is termed as cloud computing [1]. Cloud computing requires high performance and efficient underlying microarchitectures so as millions of customers (users) simultaneously can access the available resources (storage, computing, etc.) on the cloud. To gain high computing performance and throughput, multicore and multinode architectures have been devised [2, 3].

Cloud computing can be defined as a model to enable ubiquitous, convenient, and on-demand network access to a shared pool of configurable computing resources (e.g., networks, servers, storage, applications, and services), where these computing resources can be rapidly released with minimal management effort and less service provider interaction [4].

According to a survey released by North Bridge Venture Partners, in conjunction with Gigaom Research and a record 72 collaborating organizations on 19th June, 2014, 56% of businesses are using IaaS technologies to harness elastic computing resources. It is also reported that over eleven thousand cloud services and APIs (Application Program Interfaces) are currently in use by the cloud customers and the tendency is towards every-thing-as-a-service in the future (http://www.northbridge.com/cloud-computing). Similarly, according to Bezos's law, it is observed that, over the history of cloud, one unit of computing power price is reduced by 50% approximately every three years (https://gigaom.com/2014/04/19/moores-law-gives-way-to-bezoss-law). As the cloud computing price reduces, most of the enterprises will dump their data centers and move to the public cloud, thus saving money. As there will be more data traffic on public cloud clusters in the future, there is need to understand the nature, size, and type of workload in advance to efficiently manage resources for minimizing energy consumption, maintaining quality of service, and reducing cost.

IaaS cloud resources can be efficiently managed and utilized by predicting either the future workload or the future resource utilization pattern. The nature and type of workload at a public cloud are not deterministic, so some cognitive techniques are required to predict the type and nature of workload along with size and rate. Also the workload does not provide realistic information about required memory and CPU before subjecting to physical machine (host). Therefore, a better way for efficient resource management is to predict the resource utilization of all physical machines within the cloud and then allocate resources that fulfill the required predicted memory, CPU, and storage. This approach has more realistic information about physical machines than the workload prediction. In this approach, we predict the memory and CPU utilization of each physical machine. The predicted utilization of all physical machines within the cloud is accumulated at cloud manager level. Then, based on accumulated predicted utilization, the resources are allocated by the cloud manager. The predicted resources utilization tells that the future workload will require memory and CPU as predicted.

In this paper, we apply an adaptive system for resource utilization prediction of IaaS cloud. The system has two approaches for prediction; when utilization pattern is suitable for Autoregressive Integrated Moving Average (ARIMA), then this approach is applied; otherwise AR-NN is applied. The remainder of the paper consists of contemporary work, system and application models, experimental setup, performance evaluation and results, and conclusion and future recommendations.

## 2. Contemporary Work

**Figure 1:**
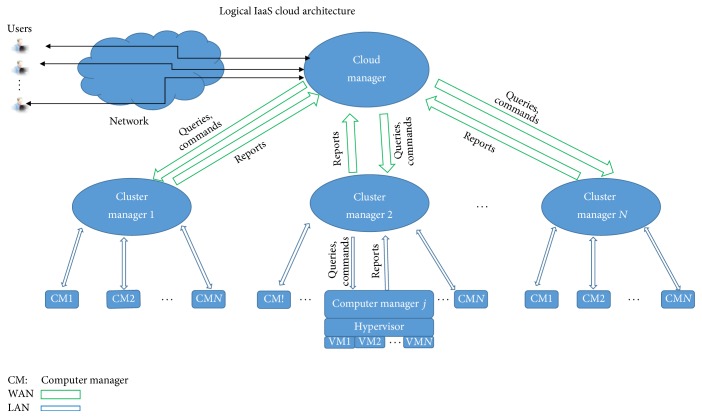
A generic IaaS cloud architecture. There may be different architectures with some more details and different positioning of logical structures. We present this simple architecture to highlight the cloud cluster and the resources it provides to customers [4, 5].

Fundamentally, each cloud computing system has the same purpose: to provide access to large pool of resources and data over internet [5]. Nurmi et al. in [5] presented an open source software framework for cloud computing: Eucalyptus. A logical architecture of Eucalyptus IaaS cloud has been shown in Figure 1. This model shows an abstract-level presentation of cloud architecture [4]. Other architectures of cloud may add some more details (components) or parallelize/split some components for performance reasons in the indicated model. IaaS cloud receives subscriber's queries/commands through cloud manager, forwarded to cluster manager and entertained through computer manager (by hypervisor and virtual machines) [5]. The operation of cloud manager, cluster managers, and computer managers has been summarized as follows:

1. Cloud manager: subscribers sign up for accounts, manage their rented resources, and access their stored data in the cloud through cloud manager. The cloud manager has mechanisms for authentication and validation of subscribers and performs top-level resource allocation. The cloud manager also enforces any cloud-global policies governing resource requests [4]
2. Cluster manager: the cluster manager manages a collection of computers connected via high-speed local area networks (e.g., 10 GB Ethernet). A cluster manager receives commands (computational tasks) and queries from the cloud manager. It checks whether part or all of a command (computational task) can be entertained by the resources of the computers in the cluster. It asks the computer manager (running on each computer in the cluster) about the availability of resources and sends back response to the cloud manager. The cluster manager then instructs the computer manager to allocate resources and reconfigures the virtual network infrastructure as per directions of the cloud manager [4]
3. Computer manager: the computer manager communicates and coordinates with the hypervisor (running on each computer in the cluster). Hypervisor receives commands from computer manager to start, stop, suspend, and reconfigure virtual machines and also to set the local virtual network configuration [4, 5]

Researchers have extensively studied resource management in cloud computing environment. We will discuss here the most relevant work to our research, due to space limitation. Silva et al. in [6] used heuristic based approach to assign resources to tasks in utility computing environment. Their study made a compromise between speedup of task execution and utilization budget of virtualized resources. Lim et al. in [7] used proportional thresholding policy for stable feedback control offered by virtual compute cloud services. However, their approach is not proactive and hence performance may degrade due to virtual machine instance creation, allocation, and initialization (booting) delay in the cloud. Thus, to overcome such performance degradation problems caused by dynamic scaling of resource, Caron et al. in [8] used past resource usage map for workload prediction. As the dynamic scaling of resources adds some overhead in case of virtual machine creation, allocation, and release, performance can be achieved if there is some prediction and then scaling mechanism in the system in response to changes in workload. In their work, they identified similar trend in past resource usage and weighted interpolation to get most similar pattern for predicting resource utilization. The predicted utilization is used as basis for making dynamic scaling decisions in real time.

Some researchers studied workload modeling and prediction techniques for capacity management and virtual machine placement in cloud environment [9–15]. Govindan et al. in [9] used statistical profiling of resources for predicting resource usage by workloads, thus minimizing energy consumption of large data centers. The prediction in these approaches is based on the statistics of workload time series [16]. Khan et al. in [17] introduced a coclustering algorithm to identify VM groups and the time periods in which certain workload patterns appear in a group [18]. They applied a multiple time series approach for workload analysis at group level rather than at individual VM level.

Some researchers have used offline or online profiling to determine application resource requirements using benchmark or real application workloads [9, 19–22]. Deriving resource requirements usually takes long time and also requires extra machine.

Recently, model-driven resource management has got enough attention from researchers. Those approaches are based on queueing theory [23] or statistical learning methods [24–27] for predicting future resource demand. In model-driven prediction, detailed prior knowledge about the problem/scenario is needed; otherwise suitable prediction results cannot be achieved. In contrast, our approach uses validity tests for selecting optimal model, so it has more diversity and acts as application- and platform-independent.

For adaptive resource allocation, some researchers used reinforcement learning [28] and control theory [29–31]. The main limitation of those methods is to specify or tune the parameters offline and add time overhead in finding the optimal solution. Rolia et al. perform dynamic resource allocation using an estimated burst factor times the most recent resource demand [32]. Gmach et al. in [33] used Fourier transform to extract long-term repeated patterns of workload. Sparse periodic autoregression for load prediction was used by Chen et al. in [34]. The problems in their approach are long prediction intervals and requirement of prior knowledge about the repetition period. Autoregression and histogram based workload prediction algorithms have been proposed in [35]. An integrated workload placement technique (i.e., demand based workload assignment along with feedback control guided workload migration) has been studied in [36]. The authors in [37] used autocorrelations to extract repeating patterns for identifying performance abnormalities. A gossip protocol for solving the load balancing problem in cloud systems has been proposed in [38]. PRESS in [39] predicts workloads by using pattern matching and state-driven approaches. Repeating patterns (signatures) are first identified by signal processing techniques. If no repeating patterns are found, then a statistical state-driven approach is applied, which uses a discrete time Markov chain for predicting future workload.

In comparison, our approach uses a combination of ARIMA and AR-NN. ARIMA model has the ability to represent different types of time series in a flexible way. Also, when used in combination with Box-Jenkins process, it can choose an optimal model for the targeted time series [40]. Due to its simplicity in terms of computation time, it can provide fast predictions in comparison to Artificial Neural Network (ANN), Markov chain, and Support Vector Machine (SVM) based approaches [41]. So it will add less prediction overhead, as required by real-time autoscaling of cloud resources. Calheiros et al. in [42] used ARIMA model in combination with Box-Jenkins process for workload prediction of Software as a Service (SaaS) cloud platform [43]. Their approach uses workload arrival rate as input to their adaptive cloud provisioning model, but we use physical resources utilization as input to our model. Tran et al. in [44] used ARIMA model for server workload prediction that targets long-time prediction, that is, up to 168 hours. Our system has the flavors of both ARIMA and AR-NN for prediction.

Workload prediction for adaptive provisioning of resources does not provide better results when compared to adaptive provisioning based on resource utilization. Workload does not provide its memory and CPU utilization; it only gives information about its data rate. By data rate one can deduce that system will receive this amount of data, but it does not provide information about how much CPU and memory it will use. Our approach explicitly predicts utilization of resources and then adaptively scales the resources that are suitable for autoscaling of IaaS cloud cluster resources.

## 3. System and Application Models

**Figure 2:**
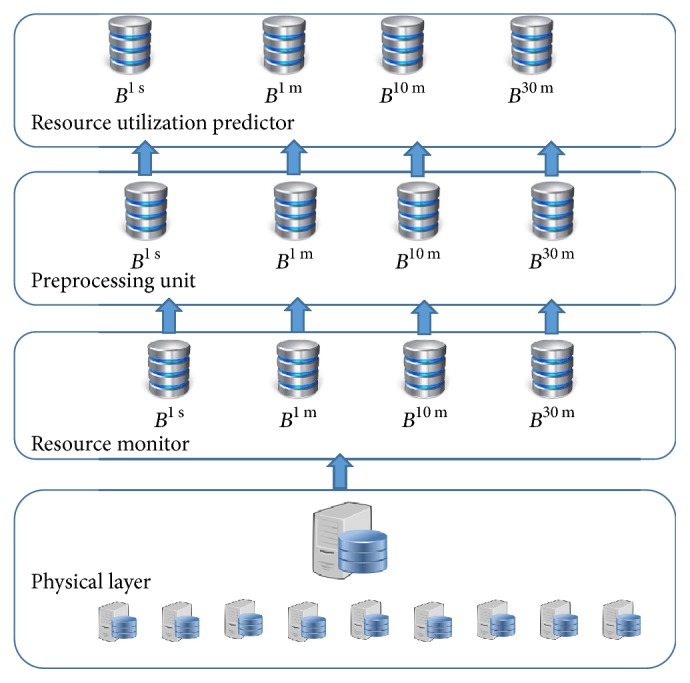
Architecture for adaptive resource utilization prediction system.

**Figure 3:**
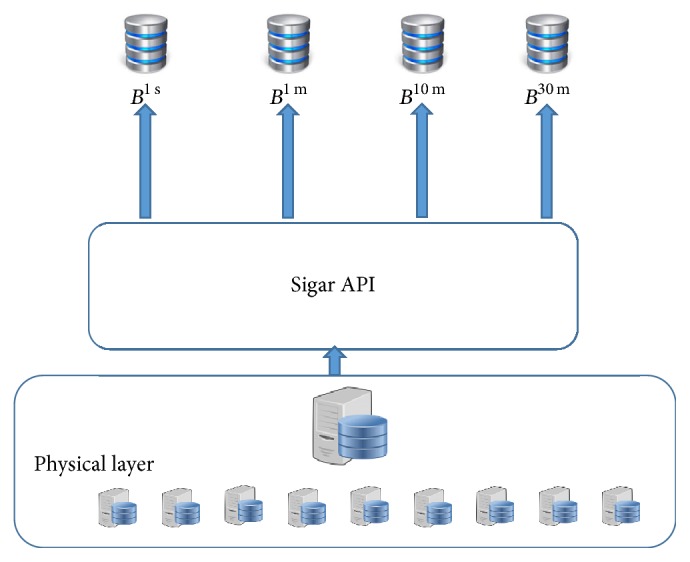
Resource monitor.

**Figure 4:**
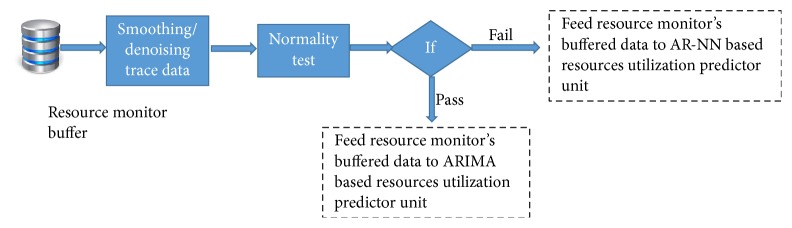
Preprocessing unit.

**Figure 5:**
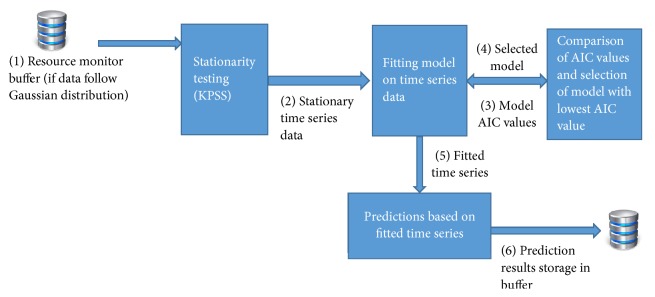
ARIMA based resources utilization predictor unit.

**Figure 6:**
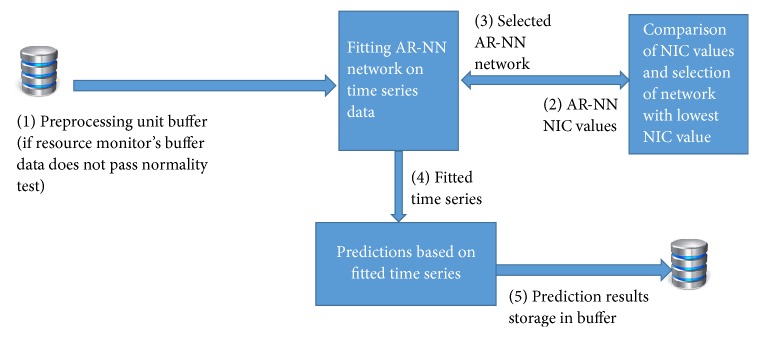
AR-NN based resources utilization predictor unit.

The proposed system model of architecture in this paper is a public cloud provider that provides resources as a service to its users (Figure 2). The system receives resource utilization (memory and CPU) history from physical layer and accumulates all physical machines' historic utilization at virtualization (IaaS) layer. The main components of our system are (1) resource monitor, (2) preprocessing unit, (3) ARIMA based resource utilization predictor, and (4) AR-NN based resource utilization predictor. The resource monitor collects utilization data of resources; the collected data is fed to preprocessing unit for checking normality. If the data set passes normality test, ARIMA is applied; otherwise AR-NN is applied. The detailed description of architecture resource monitor is shown in Figure 3. Detailed architectures of overall system, resource monitor, preprocessing unit, and resources utilization predictors are presented in Figures 2–6, respectively.

### 3.1. Resource Monitor

Our resource monitor consists of Sigar API (https://github.com/hyperic/sigar) that collects utilization of resources on different time spans. We collect utilization on each second, one minute, ten minutes, and thirty minutes of a resource. The utilization collected on each second is stored in a buffer B^1 s^, one-minute-based utilization is stored in B^1 m^, ten-minutes-based utilization is stored in B^10 m^, and half-hourly utilization is stored in B^30 m^. Data in B^1 s^ is stored for one hour, which becomes three thousand and six hundred data samples. From this we predict utilization for next one minute (i.e., 60 samples). The buffer B^1 m^ stores data for one day (i.e., 1440 samples) which is used to predict next ten minutes' utilization (i.e., 10 samples). The buffer B^10 m^ stores data for one week (i.e., 1008 samples) and is used to predict next hour utilization pattern (i.e., 6 samples). Similarly, the buffer having half-hourly collected data stores data for one month (i.e., 1440 samples) and is used to predict next day utilization (i.e., 48 samples). Thus, to predict next minute utilization, buffer B^1 s^ is used, for next ten minutes' predictions, B^1 m^ is used, for next hour, B^10 m^ is used, and for next day, B^30 m^ is used. The selected buffer is read by preprocessing unit for testing normality and transformation purposes.

### 3.2. Preprocessing Unit

Physical machines usage time series are smoothed by some filtering techniques. We use simple moving average (SMA) filter for smoothing machines usage time series which is given as follows:

(1) $$\begin{array}{c} y\left(\;i\;\right)=\frac{1}{M}\overset{M-1}{\underset{j=0}{\sum }}x\left(\;i+j\;\right), \end{array}$$

where *x*() is input series, *y*() is output series, and *M* is the number of points in the average. As ARIMA is applicable to data sets that follow Gaussian distribution, we check normality of the data set by using Jarque-Bera test with significance level of 5% [45]. If the data set fails, the normality test and neural network based prediction is performed on the data set; otherwise ARIMA based prediction is performed as shown in Figure 4. Further we explain our resource utilization predictors.

### 3.3. ARIMA Based Resources Utilization Predictor

We interface the R statistical language with Java through rJava (http://www.rforge.net/rJava) package for real-time prediction of resources. The resources utilization predictor uses the auto.arima() function in forecast package of R statistical language. The auto.arima() function selects the best fit ARIMA prediction model based on lowest Akaike Information Criterion (AIC) and Bayesian Information Criterion (BIC) values. The selected model first fits the data and then predicts the next CPU and memory utilization values. In our approach, the resource utilization is predicted for the next time interval so as to scale resources accordingly. In this paper, we use an Autoregressive Integrated Moving Average (ARIMA) model to solve the resource utilization prediction problem as shown in Figure 5.

The Autoregressive Integrated Moving Average model is one of the econometric models widely used for time series analysis [42]. It is used to remove nonstationarity in the time series data by differencing method. It then applies Autoregression (AR) and Moving Average (MA) techniques collectively to the time series. Generic form of Autoregressive Integrated Moving Average (ARIMA) model is given as follows [46, 47]:

(2) $$\begin{array}{c} {\hat{y}}_{t}=\mu +\phi_{1}y_{t-1}+\cdots +\phi_{p}y_{t-p}-\theta_{1}e_{t-1}-\cdots -\theta_{q}e_{t-q}, \end{array}$$

where *μ* is the constant (intercept), *ϕ*_*p*_ is the Autoregression (AR) coefficient at lag *p*, *θ*_*q*_ is the Moving Average (MA) coefficient at lag *q*, and $e_{t-q}=y_{t-q}-{\hat{y}}_{t-q}$ is the forecast error observed at period *t* − *q*. In our scenario, ${\hat{y}}_{t}$ is the predicted resource utilization at time *t* and *y*_*t*−*q*_ is resource utilization of past *p* samples.

It is necessary for a time series to be transformed into a stationary time series. Let *y*_*t*_ represent the data sample at time *t* and then after a time interval *τ* let the next data sample be *y*_*t*+*τ*_. Thus the mean and variance of *y*_*t*_ and *y*_*t*+*τ*_ must be constant and independent of *t* and the autocovariance between *y*_*t*_ and *y*_*t*+*τ*_ should only be influenced by *τ* for the series to be stationary. For this purpose, ARIMA differentiates the original series until the stationary time series is achieved and constitutes *d* parameter of ARIMA(*p*, *d*, *q*). The *p* and *q* values of ARIMA(*p*, *d*, *q*) can be determined by analyzing partial autocorrelation and autocorrelation plots of the time series data, respectively. During model fitting process, selecting high order of *p* and *q* will result in very small amount of white noise variance. The prediction errors of such model will be large due to overfitting. The parameters estimation errors will be large for high-order model, so it is necessary to introduce some penalty factor to avoid fitting sample data to high-order models. Based on penalty factor, many criteria have been proposed in literature; widely used criteria are combination of AIC and BIC [48]. The AIC statistic is defined as

(3) $$\begin{array}{c} AIC\left(\;\beta \;\right)=-2\ln L_{X}\left(\;\hat{\beta },{\hat{\sigma }}^{2}\;\right)+2\left(\;p+q+1\;\right), \end{array}$$

where *β* is the coefficient vector and *σ*^2^ is the white noise variance; those values of *p* and *q* for the fitted model are selected, which minimize AIC(*β*). Also $L_{X}(\hat{\beta },{\hat{\sigma }}^{2})$ is the maximum likelihood function, where $\hat{\sigma }$ and $\hat{\beta }$ are likelihood estimators of parameters *β* and *σ* which maximize *L* for given data set *X*.

The AIC statistic has tendency towards overfitting the model order, which can be corrected by using the BIC statistic [48]. BIC statistic is defined as

(4) BIC=n−p−qln⁡nσ⌢2n−p−q+n1+ln⁡2π+p+qln⁡∑t=1nXt2−nσ⌢2p+q,

where *n* represents the number of data samples, *X* is the data set, and ${\overset{⌢}{\sigma }}^{2}$ is the maximum likelihood estimate of the white noise variance.

The time series is first converted into stationary time series by differentiation, which constitutes parameter *d* of ARIMA process. Then, from (3) and (4), those values of *p* and *q* are selected, which minimize AIC and BIC statistics. Thus the fitted model is used for predicting future utilization values of memory and CPU as given in (2).

### 3.4. AR-NN Based Resources Utilization Predictor

As discussed in previous section, the R statistical language is linked with Java through rJava (http://www.rforge.net/rJava) package for real-time prediction of resources. Here we use an Autoregressive Neural Network that uses lagged values of time series as input, as shown in Figure 6. Our AR-NN (Autoregressive Neural Network) has three layers, that is, an input layer, one hidden layer, and an output layer. We use Network Information Criterion (NIC) for selecting the optimal network model for the given training data set [49].

#### 3.4.1. Autoregressive Neural Network (AR-NN)

Autoregressive Neural Network (AR-NN) is a suitable candidate for nonlinear time series forecasting. In comparison with strong forecasting models like ARIMA, the AR-NN models have shown better performance [50]. A generic *n*-lagged AR-NN model having *h* hidden neurons can be represented as follows:

(5) $$\begin{array}{c} y_{t}=a_{0}+\overset{n}{\underset{i=1}{\sum }}a_{i}y_{t-i}+\overset{h}{\underset{j=1}{\sum }}g\left(\;\omega_{0j}+\overset{n}{\underset{i=1}{\sum }}\omega_{ij}y_{t-i}\;\right)\beta_{j}+\varepsilon_{t}, \end{array}$$

where *a*_0_ is the intercept, *a*_*i*_ is vector of autoregressive coefficients, and *β* is weights vector of nonlinear part of AR-NN. The function *g*() is activation function and *ε*_*t*_ is stochastic error of the model.

Let *y*_*t*_ be the output at time *t*; then the estimated output ${\overset{⌢}{y}}_{t}$ is ${\overset{⌢}{y}}_{t}=y_{t}-\varepsilon_{t}$.

Thus

(6) $$\begin{array}{c} {\overset{⌢}{y}}_{t}=a_{0}+\overset{n}{\underset{i=1}{\sum }}a_{i}y_{t-i}+\overset{h}{\underset{j=1}{\sum }}g\left(\;\omega_{0j}+\overset{n}{\underset{i=1}{\sum }}\omega_{ij}y_{t-i}\;\right)\beta_{j}. \end{array}$$

The performance of the model can be improved by gradually minimizing the squared error:

(7) $$\begin{array}{c} {\varepsilon_{t}}^{2}={\left\|\;y_{t}-{\overset{⌢}{y}}_{t}\;\right\|}^{2}. \end{array}$$

During training session of AR-NN, all the weights and coefficients are initialized randomly, and the squared error is computed and checked to determine whether it approaches zero or not. The weights are determined by partial differentiation of (7) with respective weight. Another important consideration is the number of nonlinear units that cannot be determined by the standard error metrics like RMSE, MSE, and so forth. Extra nonlinear units add computational and space complexity to the model, so there should be a limited number of nonlinear units. As mentioned above, a better criterion is NIC, which limits the number of nonlinear units.

## 4. Experimental Setup

**Figure 7:**
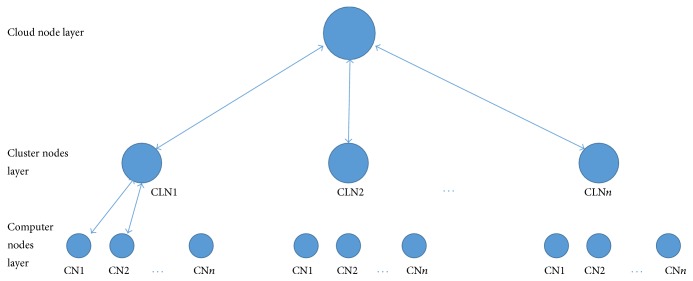
Experimental setup.

Our experimental setup consists of three layers, computer nodes layer, cluster nodes layer, and cloud node layer, as shown in Figure 7. Computer nodes represent IaaS servers that execute cloud users' virtual machines (VMs). Hundreds to thousands of computer nodes are connected to a cluster node through high-speed local area network (LAN). Each computer node reports its usage to respective cluster node. Similarly, each cluster node is connected to cloud node through wide area network (WAN). Cluster node accumulates usage of its connected computer nodes and reports to cloud node. Cloud node accumulates usages received from cluster nodes. At cloud node, periodic usage time series is subjected to preprocessing unit and then forwarded to resource utilization predictor for predicting future utilization of the cloud as explained in Section 3. We have evaluated our system with a real trace named as fastStorage (http://gwa.ewi.tudelft.nl/datasets/gwa-t-12-bitbrains) which has 1250 VMs, memory of 17729 Gigabytes, and 4057 CPU cores.

## 5. Performance Evaluation and Results

**Figure 8:**
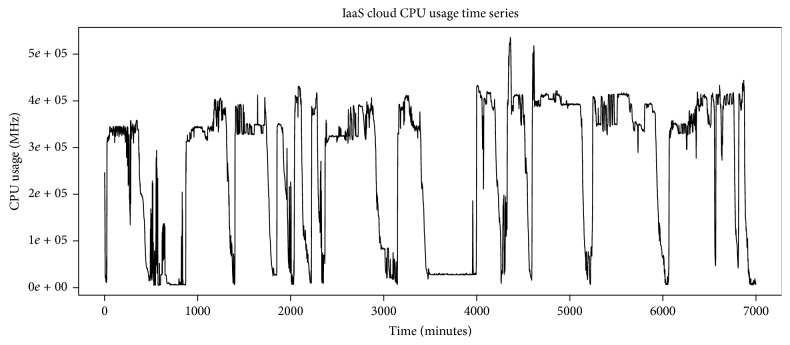
CPU usage time series of fastStorage trace.

**Figure 9:**
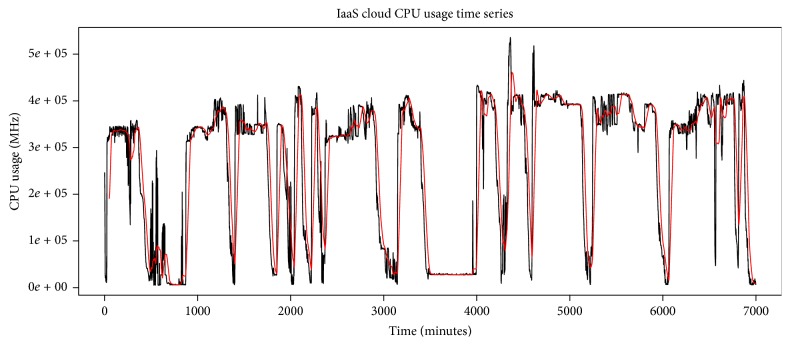
Original time series and simple moving average filtering.

**Figure 10:**
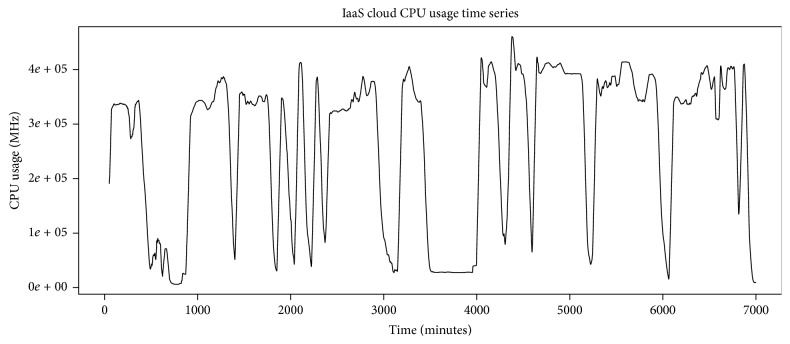
SMA filtered series with *M* = 50.

We have evaluated our system with real trace, fastStorage, recorded for 7000 minutes. The cloud has 1250 VMs that are connected to fast Storage Area Network (SAN) devices. The trace includes a random selection of VMs from Bitbrains data center. CPU usage time series of the trace has been shown in Figure 8. The trace time series has higher frequencies; so to remove these noisy higher frequencies, we apply SMA filter.  Figure 9 shows the noisy time series along with smoothed time series. The filtering result has been shown in Figure 10. We apply 50-point SMA filter, which filters out the higher noise frequencies. We then apply Jarque-Bera test to check whether the smoothed time series follows normal distribution or not. If the time series follows normal distribution, we apply ARIMA process; otherwise AR-NN is applied.

### 5.1. ARIMA Based Prediction Performance and Results

**Figure 11:**
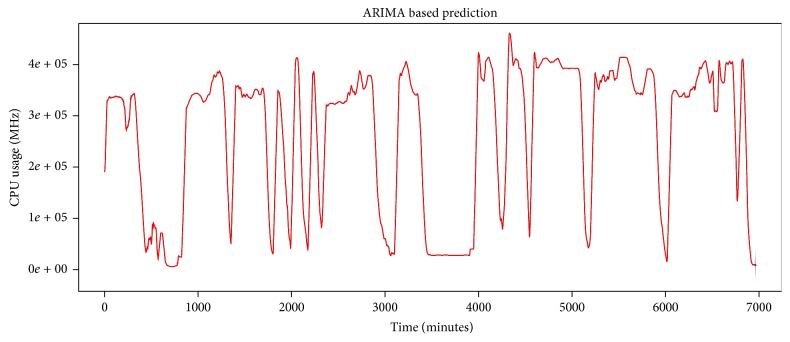
ARIMA based model fitting to IaaS cloud CPU usage time series and future usage prediction.

The data set is tested by Jarque-Bera test with significance level of 5% for normality. Test results show that the data set does not follow normal distribution. Test results have been shown in Table 1. The test result of data set shows that computed *p* value is less than alpha (i.e., 0.02); thus null hypothesis can be rejected and alternate hypothesis becomes true. Our system will select AR-NN for prediction purpose. For testing purpose, we have applied ARIMA model to the data set that generates results shown in Figure 11. Predicted results have been tested on various accuracy metrics, that is, Mean Error (ME), Root Mean Squared Error (RMSE), Mean Absolute Error (MAE), Mean Percentage Error (MPE), and Mean Absolute Percentage Error (MAPE). The fitted model's prediction errors tested on various accuracy metrics are given in Table 2. All accuracy metrics' tests show that ARIMA based prediction is not suitable for the given data set.

### 5.2. AR-NN Based Prediction Performance and Results

As stated earlier, the data set fails normality test at 5% significant level. Thus our system applies AR-NN to the data set for predicting future workload. The AR-NN applies twenty models one by one to the data set and selects the one that has the lowest NIC value. The selected AR-NN model has three layers, that is, input layer, hidden layer, and output layer. Input layer has 18 neurons, which simply take eighteen-lagged inputs, and forwards them to hidden layer. Hidden layer has 10 neurons, which perform actual processing, and forwards processed data to output neuron (i.e., output layer has single neuron).

#### 5.2.1. Training of AR-NN Model

First eighteen samples of data set are used for training the AR-NN model. The selected model has a total of two hundred and one weights; out of those, one hundred and eighty are lagged input weights that lead to hidden layer, ten are coefficient weights (i.e., one for each hidden neuron), ten are hidden layer output weights that lead to output layer, and there is one weight of output unit. All these weights are trained for training data.

#### 5.2.2. Validation Results of AR-NN Model

**Figure 12:**
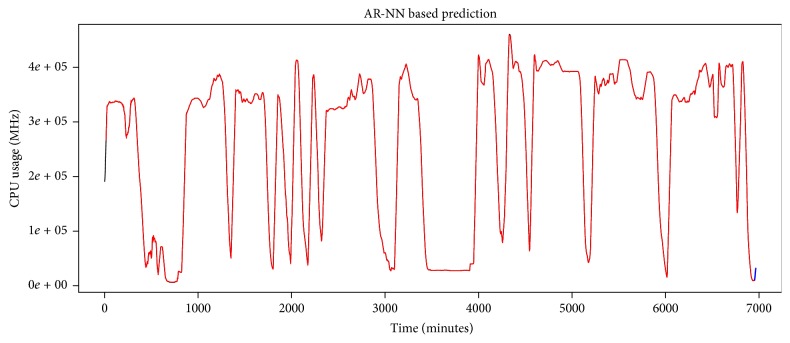
AR-NN based model fitting to IaaS cloud CPU usage time series and future usage prediction.

The AR-NN model is validated by six thousand eight hundred and eighty-three samples of data set. The validation results are shown in Figure 12, where red line shows validated/fitted results and black line in the start shows training data. As red (fitted) and black (actual) lines overlap, the model best fits the validation data.

#### 5.2.3. Prediction Results and Performance of AR-NN Model

**Figure 13:**
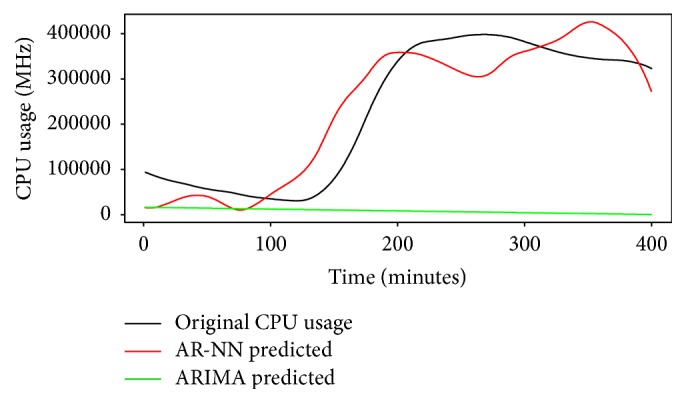
Comparison of predicted results.

The model is used to predict future four hundred minutes' usage that is shown with blue line in Figure 12. We evaluate performance of the model based on predicted results; for that we compare 400 minutes' actual data set values and predicted results based on several performance metrics tabulated in Table 2. The MAPE of predicted result is 19.08957285%, which is a better result when compared with ARIMA's prediction MAPE, that is, 6784.02403%. Actual and predicted time series have been shown in Figure 13. Our results show that, for the given data set, AR-NN performs better than ARIMA.

## 6. Conclusion and Future Recommendations

In this paper, we presented an adaptive resource utilization prediction system for IaaS cloud. The system extracts physical resources utilization, stores the utilization patterns, and checks normality; if utilization pattern passes normality test, it applies ARIMA models based on AIC values; otherwise AR-NN algorithm is applied, which selects AR-NN model based on NIC values. The model with lowest AIC or NIC value is selected for fitting the training data, which then predicts future utilization demand. We used real trace, that is, fastStorage of Bitbrains data center, for evaluating our system. The system predicts four hundred future data samples, that is, workload demand for 400 minutes. The results show that AR-NN has better results than ARIMA for a data set that fails normality test with confidence level of 5%. Also the system can use low prediction confidence limits for energy efficiency and economy of scale but high confidence limits for better quality of service to its customers. Furthermore, other prediction techniques like deep learning based networks can be used, while noticing both the temporal and spatial complexities of the method in comparison with ARIMA and AR-NN. Also hybrid techniques can be used depending on the size of training data, duration for prediction, and type of prediction patterns (i.e., a minute utilization, ten minutes' utilization, hourly pattern, daily pattern, network usage, etc.).

## Figures and Tables

**Table 1:** Jarque-Bera test results of IaaS cloud CPU usage data set.

Jarque-Bera test results	
Test metric	Value
JB (observed value)	7.967
JB (critical value)	5.991
*p* value	0.00000000000000022204
Alpha	0.02

**Table 2:** ARIMA based prediction accuracy by various metrics.

Accuracy metric	AR-NN prediction	ARIMA prediction
ME	6969.056306	−222841.1743
RMSE	61449.22523	270979.9746
MAE	51220.64295	222841.1743
MPE	−31.4578077	−6784.02403
MAPE	19.08957285	6784.02403
